# Cytotoxicity of Phenol Red in Toxicity Assays for Carbon Nanoparticles

**DOI:** 10.3390/ijms131012336

**Published:** 2012-09-26

**Authors:** Ying Zhu, Xiaoyong Zhang, Jianhua Zhu, Qunfen Zhao, Yuguo Li, Wenxin Li, Chunhai Fan, Qing Huang

**Affiliations:** 1 Laboratory of Physical Biology, Shanghai Institute of Applied Physics, Chinese Academy of Sciences, Shanghai 201800, China; E-Mails: xiaoyongzhang1980@gmail.com (X.Z.); yuguo98@hotmail.com (Y.L.); fanchunhai@sinap.ac.cn (C.F.); huangqing@sinap.ac.cn (Q.H.); 2 School of Pharmacy, Fudan University, Shanghai 200032, China; E-Mail: jhzhu@shmu.edu.cn; 3 Faculty of Life Science and Bioengineering, Ningbo University, Ningbo 315211, China; E-Mail: zhaoqunfen@nbu.edu.cn

**Keywords:** carbon blacks (CBs), multi-walled carbon nanotubes (MWNTs), cytotoxicity, phenol red, adsorption, ^125^I-labeling

## Abstract

To explore the novel properties of carbon nanoparticles (CNPs) in nanotoxicity assays, the adsorption of phenol red (a pH indicator for culture medium) by multi-walled carbon nanotubes (MWNTs) and three kinds of carbon blacks (CBs) with nanosize, and its effects on cytotoxicity were studied. Results indicated that the phenol red adsorbed and delivered into cells by CBs was responsible for the toxicity to Hela cells in the medium without serum. The cellular uptake of phenol red was verified using ^125^I-labeling techniques. The size-dependent cytotoxicity of CBs was found to closely correlate to adsorption of phenol red, cellular uptake of phenol red-CB complexes and the amount of phenol red delivered into the cells by CBs. Although the CBs were either nontoxic or slightly toxic, as vehicles of phenol red, they played an essential role in the cytotoxicity induced by phenol red. However, MWNTs showed an intrinsic cytotoxicity independent of phenol red. The implications associated with these findings are discussed.

## 1. Introduction

Increasing effort has been made to evaluate the cytotoxicity of carbon nanoparticles (CNPs). However, the results obtained are often fragmentary and even puzzling [1–6]. These inexplicable results may be related to the novel properties of CNPs. Because of their large surface area and high adsorption capability, some molecules, especially dye molecules, can be adsorbed onto CNPs, leading to the interference of cellular viability determinations based on optical absorption measurements [7,8]. Hurt *et al*. addressed this confounding issue and advised caution when performing even an established toxicity assay in the presence of fine carbon particles [4]. Besides dye molecules, Guo *et al*. studied the adsorption of essential micronutrients from cell culture media by single-walled carbon nanotubes (SWNTs) and indicated that SWNTs caused dose-dependent adsorption and depletion of amino acids and vitamins. HepG2 cells cultured in such depleted media showed significantly reduced viability [9].

However, problems are not just limited to simple adsorption by CNPs interfering with viability assays or depletion of micronutrients from the medium. It is well known that CNPs can be largely internalized into the cells [10–13]. Moreover, some chemical molecules, once adsorbed onto CNPs, can be delivered into cells. Therefore, CNPs have great potential in the development of various drug delivery systems [14–16]. It is expected that a similar situation will also occur in toxicity assays for nanoparticles. Some cargos in biological media can be vectorized into cells by CNPs and during assessing toxicity of CNPs with common toxicological methods some unexpected phenomena or results emerge (*i.e.*, the “Trojan horse” effect) [4].

In our previous work, it was indicated that serum proteins in cell culture medium adsorbed on CNPs attenuated the inherent cytotoxicity of CNPs and the extent of toxicity attenuation increased with increasing amounts of adsorbed serum proteins [13]. Recently, Ge *et al*. investigated the nanoparticle-protein interactions in detail and found that binding of blood proteins to carbon nanotubes greatly altered their cellular interaction pathways and resulted in much reduced cytotoxicity for these protein-coated nanoparticles, according to their respective adsorption capacity [17].

Regarding the adverse bio-effects, we are wondering whether there are some harmful components in cell culture medium that play essential roles with respect to the toxicity after being transported into cells by CNPs. Our latest work indicated that a large amount of sodium ions in serum-free medium were adsorbed and delivered into the cell interior by nanodiamonds, which then led to severe cytotoxicity [18]. In this work, the interaction of CNPs with phenol red, a pH indicator for culture medium, and its effects on cytotoxicity were studied. During examination of the cytotoxicity of CNPs, surprisingly, we found for the first time that phenol red, which has been widely employed in *in vitro* toxicological assays, was toxic to Hela cells. Using radioactivity tracer technology, the toxicity mechanism of phenol red was verified.

## 2. Results and Discussion

### 2.1. Characterization of CNPs

**Figure 1:**
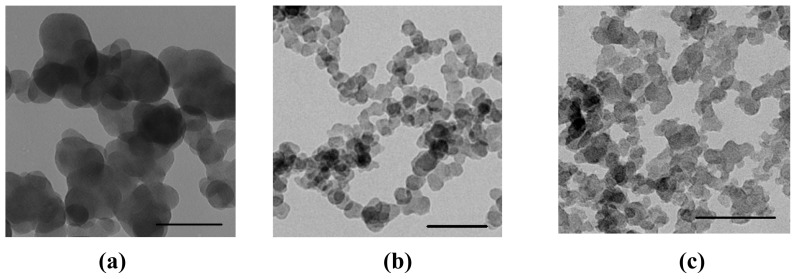
TEM images of carbon black (**a**) PG; (**b**) S160; (**c**) P90. Scale bar = 100 nm.

Three kinds of carbon black (CB), CB PG, CB S160, and CB P90 are denoted by PG, S160, and P90, respectively. TEM images show that they have primary particle sizes of 51, 20, and 14 nm for PG, S160, and P90, respectively (Figure 1).

The diameter of MWNTs used in this work is about 40–100 nm. After purification, cutting and functionalization by oxidation and sonication, well-dispersed MWNTs with about 600–800 nm in length were prepared. Elemental analysis by inductively coupled plasma mass spectrometry (ICP-MS) indicated that the MWNTs contained impurities of 0.1% Ni and 0.2% Fe. The details for characterization have been described in our previous work [19].

### 2.2. Adsorption of Phenol Red on CNPs

**Figure 2:**
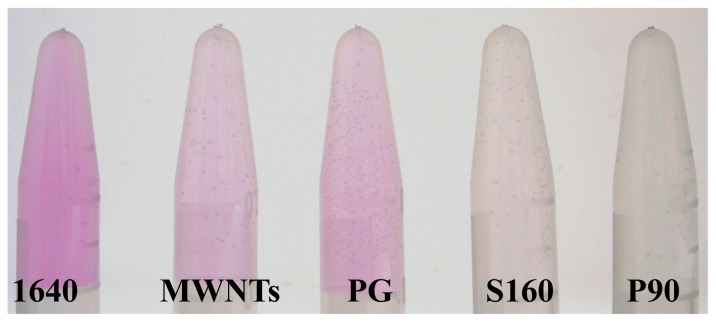
The color change of phenol red in supernatants of carbon nanoparticles (CNPs) after 2 h incubation in serum-free medium.

Several researchers have indicated that phenol red can be adsorbed onto SWNTs, leading to attenuation of their UV/Visible spectroscopic characteristics [20,21] or the removal of their characteristic color by centrifugal ultrafiltration [9]. However, no reports exist thus far on the effects of adsorption of phenol red by SWNTs on living systems. To visualize the adsorption of phenol red on CNPs, MWNTs and three kinds of CB were incubated in RPMI1640 medium without serum for 2 h. After centrifugation, the characteristic pink color of phenol red was attenuated to varying degrees (Figure 2). To quantify the adsorption, the amounts of phenol red adsorbed on each kind of CNPs were determined in media with and without serum, and the results listed in Table 1.

Upon decreasing the size of the CNPs, we observed a monotonic increase in the amounts of adsorbed phenol red. The extremely high adsorption ability of P90 led to the formation of a colorless medium without an associated change in pH value. The MWNTs had an intermediate adsorption ability located between those for PG and S160. The difference in adsorption abilities of CNPs was in consistent with the sequential order of change in color of phenol red, shown in Figure 2. However, relatively lower adsorption abilities were obtained for all CNPs in culture medium with serum, implying inhibition of the adsorption of phenol red on CNPs by competitive adsorption from the serum proteins.

### 2.3. Effects of Phenol Red on Cytotoxicity

**Figure 3:**
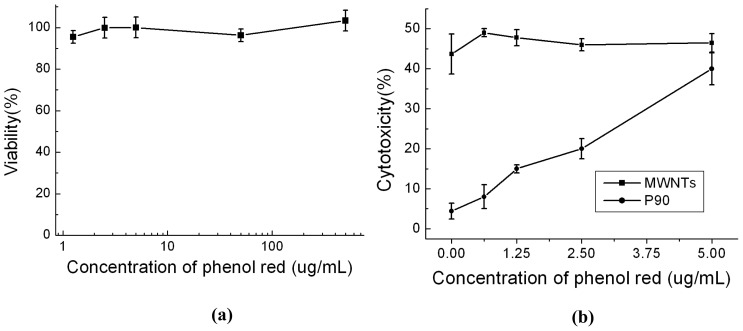
Dose-dependent cytotoxicity of phenol red in serum-free medium (**a**) without CNPs; (**b**) with CNPs at concentration of 100 μg mL^−1^. All data shown are based on three independent experiments.

In the general sense, phenol red should be nontoxic to mammalian cells. Indeed, no toxicity was observed in this work when Hela cells were incubated even in a RPMI1640 solution containing up to 500 μg mL^−1^ phenol red (Figure 3a), which is one hundred times higher than the normal concentration of 5 μg mL^−1^ in complete cell culture medium. When P90 existed in the culture medium, however, the effect of phenol red on cytotoxicity appeared. As illustrated in Figure 3b, over the concentration range of phenol red used, we found a monotonic increase in cytotoxicity with increasing concentration of phenol red, with no toxicity showed in medium without phenol red. Thus it can be seen that phenol red was responsible for the cytotoxicity, and its toxicity appeared only in the presence of P90 in the culture medium. Unlike P90, the MWNTs exhibited an obvious cytotoxicity over the entire concentration range of phenol red (Figure 3b), and the cytotoxicity remained essentially unchanged at phenol red concentrations from 0 to 5 μg mL^−1^.

**Figure 4:**
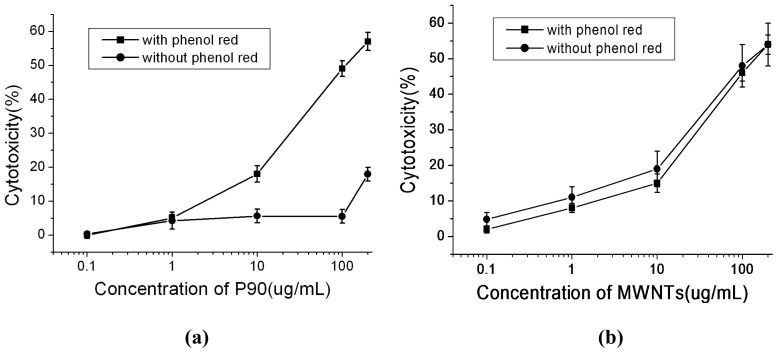
The cytotoxicity of CNPs dispersed in serum-free medium with or without phenol red (**a**) P90; (**b**) MWNTs. All data shown are based on three independent experiments.

Having observed the cytotoxicity of phenol red associated with CNPs, we next examined the dose-dependent toxicities of MWNTs and P90 in culture media with and without phenol red. As seen in Figure 4a, when Hela cells were exposed to P90 in culture medium without phenol red, no noticeable toxicity was found until the concentration of P90 reached up to 200 μg mL^−1^. However, in culture medium containing phenol red, the cytotoxicity continuously increased with increasing P90 concentration. Unlike P90, MWNTs exhibited increasing cytotoxicity with increasing concentration of MWNTs no matter whether phenol red existed in the medium or not (Figure 4b).

**Figure 5:**
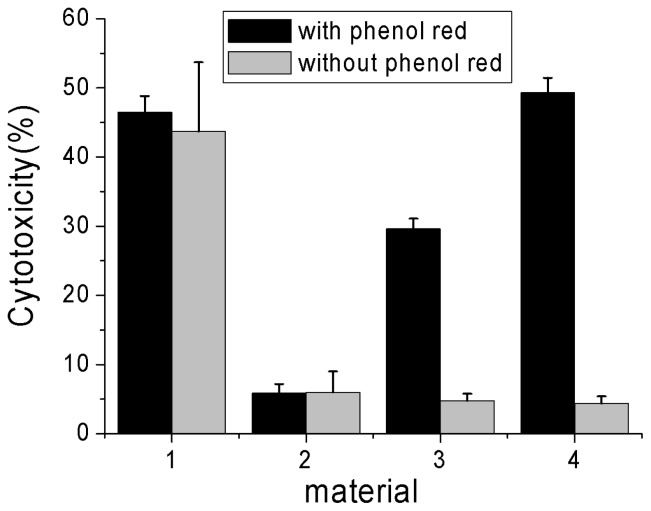
Cytotoxicity assessment of Hela cells exposed to MWNTs, PG, S160 and P90 (denoted by 1, 2, 3 and 4, respectively) in serum-free medium with or without phenol red. All data shown are the mean values of three experiments ± SEM.

Figure 5 shows the toxicities of 100 μg mL^−1^ PG, S160, P90, and MWNTs to Hela cells in culture medium with and without phenol red. One can see that three kinds of CBs exhibited different toxicity in media with phenol red. Moreover, a distinct size-dependent cytotoxicity appeared, with the finest P90 being most toxic. When phenol red was absent in the media, however, the different cytotoxicity of CBs disappeared almost completely. A further trypan blue exclusion test confirmed this MTT-based cytotoxicity assessment results (Figure S1). All of these results suggest that phenol red exhibited no toxicity because of its poor cellular penetration, and the cytotoxicity observed in the presence of CB would be due to the production of cell-penetrating phenol red-CB complexes. CB could be either toxic or nontoxic, depending on whether phenol red existed in the medium or not.

Our previous work indicated that CBs can be largely internalized into Hela cells in a serum-free medium [13], in combination with Figure 5, we concluded that all of these CBs in culture media without phenol red were nontoxic even after being internalized into the cells. In contrast, there was no noticeable difference in cytotoxicity when the cells were exposed to MWNTs in culture medium with or without phenol red and MWNTs exhibited pronounced cytotoxicity in the culture medium no matter whether phenol red existed in medium or not (Figure 5). This led us to conclude that MWNTs themselves were potential environment and health hazards.

In addition, CBs still exhibited a certain cytotoxicity when cells were incubated in complete cell culture medium (see Figure S2). In this case phenol red was not responsible for the toxicity due to low absorption ability. It is suggested that some unknown harmful substances in medium might be adsorbed and delivered into cells by CBs, leading to cell death. Further study related with the additional toxicity mechanism is required.

### 2.4. Cellular Uptake of Phenol Red and Its Dependence on Size of CBs

In this work, a distinct size-dependent cytotoxicity of CBs dispersed in culture medium containing phenol red was found (Figure 4). The study of the effects of nanoparticle size on cytotoxicity is an attractive topic in nanotoxicology [22–24]. Generally speaking, researchers agree that smaller particles are more toxic than larger particles [25–27]. However, the scientific nature behind these phenomena is unclear. As phenol red is an origin of toxicity, it is reasonable to speculate that the degree of toxicity should be decided by the quantity of phenol red delivered into cells by CBs. Hereby the exploration of reasons for size-dependent toxicity observed in this work may be helpful to better understand the toxicological mechanisms.

In our previous study with ^99m^Tc radiolabeling method, the size-dependent cellular uptake of CBs has been demonstrated [13]. In the present work, the size-dependent adsorption amounts of phenol red by CBs have also been demonstrated (Table 1). On the basis of these results, the size-dependent amounts of phenol red delivered into cells by CBs, should logically be valid. As a result, the size-dependent toxicity of CBs to Hela cells is reasonably explained.

To experimentally verify this speculation, phenol red was then labeled with the radionuclide ^125^I via a conventional Iodogen method [28]. The stability of the ^125^I-phenol red was very high and its radiochemical purity was maintained over 99% even after one month (Figures S3, S4). The amounts of phenol red internalized into the cells were determined to be 1.68, 7.10, and 25.8 ng per 1000 cells for PG, S160, and P90, respectively (Table 2). When comparing the cellular uptake of 0.31 ng for the control determined in cell culture medium without CBs, phenol red was indeed transferred into the cells by CBs. The experimental uptake values demonstrated the size-dependence of the amounts of phenol red delivered into the cells, with the finest P90 had highest uptake of phenol red. More importantly, the results supported our speculation that the size-dependent cytotoxicity was based on the size-dependence of the cellular uptake of CB complexes and the amounts of phenol red adsorbed by CBs.

Finally, it should indicate that toxicity of phenol red was exhibited only when cells were exposed to CBs in medium without serum. As seen from Table 2, when cells were exposed to the finest P90, the existence of serum in culture medium reduced the uptake of phenol red from 25.8 ng to 0.52 ng per 1000 cells. As a result, a little phenol red internalized into cells will not interfere with toxicity assessments of CBs obtained in complete cell culture medium (Figure S2), implying that the assessments of cytotoxicity for CNPs, in most cases with complete cell culture medium, are reliable. During the study on drug delivery systems of CNPs, especially for gene delivery, however, the cell culture medium without serum aimed to increase the efficacy was preferentially used [29,30]. Accordingly caution is needed against the influence of nanopaticle-phenol red interactions on efficacy of nanoparticle-based drug delivery systems.

## 3. Experimental Section

### 3.1. Carbon Nanoparticles (CNPs)

Three kinds of CB with primary particle sizes of 51, 20, and 14 nm for PG, S160, and P90, respectively, were purchased from Degussa (Shanghai, China). All of these nanoparticles were used directly in the experiments without any pretreatment.

MWNTs (95% in purity and several tens of micrometers long with diameter of 40–100 nm) prepared by chemical vapor deposition (CVD) were obtained from Shenzhen Nanotech Port Co. Ltd, China. After purification, cutting and functionalization by oxidation and sonication, well-dispersed MWNTs with about 600–800 nm in length were prepared. Before experiment, their characterization had been completed by using various microscopic techniques. The details for preparation and characterization have been described in our previous work [19].

### 3.2. Standard Curve Drawing of Phenol Red in Culture Medium

A certain amount of phenol red was dissolved in RPMI-1640 without phenol red (RPMI-1640W, Gibco), to give suspensions with a concentration of 1 mg mL^−1^. The resulting solution was diluted into a series of working solutions with phenol red of gradient concentration, 10 μg mL^−1^, 5 μg mL^−1^, 2.5 μg mL^−1^, 1.25 μg mL^−1^, 0.625 μg mL^−1^ and 0.313 μg mL^−1^. The adsorption feature of phenol red was detected at 560 nm using a UV/Vis spectrophotometer (TU-1800SPC, China), and RPMI-1640W was used as the reference solution [20,21]. The standard curve of phenol red in culture medium was given in Figure S5.

### 3.3. Determination of Adsorption Amounts of Phenol Red on CNPs

Various CNPs were added into cell culture medium (RPMI-1640, containing 5 μg mL^−1^ phenol red, Gibco), with and without serum, to give suspensions with a final concentration of 1 mg mL^−1^. After sonication, the resulting suspensions were incubated at 37 °C for 2 h. Next, the suspensions were centrifuged at 12,000 rpm for 10 min, and the concentration of phenol red in supernatants for all samples, as well as the original cell culture medium, were measured using the same method as described above. The adsorption amounts of phenol red on CNPs per milligram, *q* (μg mg^−1^), were calculated for various CNPs according to Equation 1:

(1) $$q=\frac{Co-Ce}{Cn}$$

where *C_o_* and *C_e_* are the phenol red concentrations (μg mL^−1^) contained in the original cell culture medium and in the supernatants, respectively. *Cn* is the concentration of CNPs in the cell culture medium (mg mL^−1^).

### 3.4. Assay for Phenol Red Concentration-Dependent Viability of Cells in Serum-Free Medium

A stock solution of 1 mg mL^−1^ phenol red was prepared by the addition of certain amounts of phenol red (Amresco) into RPMI-1640W. Various culture media with phenol red concentrations of 0, 1.25, 2.5, 5.0, 50 and 500 μg mL^−1^ were obtained by dilution of the stock solution with RPMI-1640W. Hela cells were grown in a normal complete cell culture medium, and the resulting cell suspension (10^5^ cells mL^−1^) was dispensed into 6-well plates and incubated overnight to allow for cell adherence. After washing twice with phosphate buffered saline (PBS), the cells were incubated in serum-free medium with phenol red at various concentrations. The cells in the normal serum-free medium containing 5 μg mL^−1^ phenol red were used as controls. All samples were incubated at 37 °C for 2 h, and then washed three times with PBS. The cells were further incubated for 24 h in complete cell culture medium. Finally, the cell viability was determined by the [3-(4,5-dimethylthiazol-2-yl)-2,5- diphenyltetrazolium bromide] MTT assay (Sigma-Aldrich, Shanghai, China) and expressed as a percentage of OD_treat_/OD_control_. All viability determination data were based on three independent experiments.

### 3.5. Assay for Concentration-Dependent Cytotoxicity of Phenol Red in Serum-Free Medium with CNPs

To verify the effects of formation of CNP–phenol red complexes on cytotoxicity, a special procedure, similar to that described [13,31], was used for the cytotoxicity assays. Various culture media with phenol red concentrations of 0, 0.625, 1.25, 2.5 and 5.0 μg mL^−1^ were prepared using a similar method to that described above. Certain amounts of MWNTs and P90 were added into each medium to yield a nanomaterial concentration of 100 μg mL^−1^. Cells incubated in serum-free medium without CNPs were used as a control. After 2 h incubation at 37 °C, all samples were washed four times with PBS to remove the uninternalized CNPs, and then further incubated in complete cell culture medium for 24 h. Cytotoxicity in the culture medium with phenol red and CNPs was determined by a similar method as described above. The cell viability was expressed as a percentage of OD_nanomaterial_/OD_control_, and the relative cytotoxicity was calculated according to the formula: (1−OD_nanomaterial_/OD_control_) × 100. All cytotoxicity assessment data were based on three independent experiments.

### 3.6. Assay for Cytotoxicity of CNPs in Serum-Free Medium with or without Phenol Red

Stock solutions of test nanomaterials were prepared by the addition of CNPs into RPMI-1640 solution with or without phenol red. The test CNP stock solutions were then added into the same medium as the cells to make the final required concentrations of CNPs. The cells in the same culture solution without CNPs were used as controls. After 2 h incubation, all samples were washed with PBS and further incubated in complete cell culture medium for 24 h. The cytotoxicity of the CNPs in the serum-free medium with or without phenol red was assessed using the MTT method.

Trypan blue exclusion test was used to distinguish the live and dead cells. After incubation, cell aliquots were collected and immediately stained with trypan blue for 5 min. Cell proliferation was measured by counting cell numbers and cell viabilities were expressed as a percentage of Number_nanomaterial_/Number_control_, and the relative cytotoxicity was calculated according to the formula: (1−Number_nanomaterial_/Number_control_) × 100. All cytotoxicity assessment data were based on three independent experiments.

### 3.7. ^125^I Labeling of Phenol Red

The radiolabeling of phenol red was performed by the conventional Iodogen method [28]. Briefly, a solution of phenol red at a concentration of 30 μg mL^−1^ was added to a vial coated internally with Iodogen (Chengdu Gaotong Isotope Co., China). The radiolabeling of phenol red was initiated by the addition of 18 MBq (≈500 μCi) Na^125^I and the mixture was gently mixed and incubated for 5 min at room temperature. Finally, Na_2_SO_3_ solution was added to stop the labeling reaction. A gradient high-performance liquid chromatography (HPLC) system was used for examination of the radiolabeling rate and radiochemical purity. The reserve times were 13.99 min and 2.84 min for phenol red labeled with ^125^I and free iodine ions, respectively (Figure S3). The labeling yield was determined to be 100% with no free iodine ions detected (Figure S4). The specific activity was calculated as the activity per unit amount of labeled compound and was found to be 0.6 MBq μg^−1^. The *in vitro* stability of ^125^I-phenol red was determined by incubating the labeled compound in cell culture medium at 37 °C. The aliquots were then analyzed at time intervals of 1, 2, 4 and 8 h in the manner described above (see Figure S6).

### 3.8. Determination of Cellular Uptake of Phenol Red with Radiotracer Techniques

The culture medium with ^125^I-phenol red (^125^I-medium) was prepared by addition of a certain amount of ^125^I-phenol red with high specific activity (0.6 MBq μg^−1^) into RPMI1640 solution with or without serum. Various CNPs were added into the ^125^I-medium to make the concentration of the nanomaterial stock solution up to 200 μg mL^−1^, followed by sonication just prior to the experiments. Hela cells were pretreated as described above. After washing twice with PBS, the CNP stock solutions in the ^125^I-media with or without serum were added to the plate. The final concentration of CNPs for all samples was 100 μg mL^−1^. The cells in the same ^125^I-medium without CNPs were used as controls. After 2 h incubation at 37 °C, all samples were washed four times with PBS to remove the non-internalized CNPs and ^125^I-medium. The cells were then digested with 0.25% trypsin, and re-suspended in 1.05 mL of the complete cell culture medium. Aliquots of 50 μL were taken from the treated samples to determine the number of cells, and the remainder of the cell suspensions was collected in centrifuge tubes to determine the activity of ^125^I-phenol red taken up by the cells. 100 μL stock solutions of ^125^I-media with or without serum were then taken as references, and counted simultaneously for the determination of the total activity of ^125^I-phenol red to which the Hela cells were exposed. The uptake quantities of phenol red per 1000 cells (ng/1000 cells) were calculated according to Equation 2:

(2) $$\text{Uptake quantities}/1000\text{cells}=\frac{Rc\times C}{Rs\times 10\times n}\times 1000\times 1000$$

where *Rc* is the activity of the cell suspension (count per minute; cpm), *Rs* is the activity of the reference samples (cpm), *n* is the number of cells per milliliter in each sample, and the concentration of phenol red in cell culture medium (*C*) is 5 μg mL^−1^.

## 4. Conclusions

This paper studied the interaction of phenol red in culture medium with CNPs and its effects on cytotoxicity. The results indicated that phenol red, commonly employed in toxicological study, was toxic after being adsorbed and delivered into cells by CBs in a serum-free cell culture medium. CBs were nontoxic themselves even after internalized into cells from serum–free culture medium without phenol red. However, as carriers of phenol red, CBs played an essential role in the cytotoxicity induced by phenol red. The findings in this work suggested that, in a departure from the study in common toxicology, some constituent components in cell culture medium, whose biological activity could be negligible in conventional toxicity assays would interfere with toxicity assessments in nanotoxicology.

## Supplementary Materials



## Figures and Tables

**Table 1:** The amounts of phenol red adsorbed on CNPs (μg mg^−1^). All data shown are based on three independent experiments.

Medium composition	CNPs			

	MWNTs	PG	S160	P90
Without serum	2.36 ± 0.26	1.43 ± 0.16	3.81 ± 0.04	4.85 ± 0.02
With serum	All data < 1.32			

**Table 2:** Cellular uptake of ^125^I labeled phenol red in cell culture medium with or without serum. The concentration of CNPs in all samples was 100 μg mL^−1^, (−: CNPs dispersed in serum-free medium; +: CNPs dispersed in complete medium). All data shown are the mean values of three experiments ± SEM.

Nanomaterials	Uptake quantities (ng 1000 cell^−1^)
Control	0.31 ± 0.07
MWNTs(−)	2.33 ± 1.10
PG(−)	1.68 ± 0.36
S160(−)	7.10 ± 1.47
P90(−)	25.8 ± 1.9
MWNTs(+)	0.38 ± 0.06
PG(+)	0.33 ± 0.08
S160(+)	0.40 ± 0.15
P90(+)	0.52 ± 0.17
