# Supplementary material for: Cytotoxicity of Phenol Red in Toxicity Assays for Carbon Nanoparticles
Source: Int J Mol Sci. 2012 Sep 26;13(10):12336–48. doi: 10.3390/ijms131012336 (PMC3497275; doi:10.3390/ijms131012336)
Supplement: Supplementary file 1 [file ijms-13-12336-s001.pdf]

Supplementary Information

Figure S1. Cytotoxicity assessment of Hela cells exposed to MWNTs, PG, S160 and P90 (denoted by 1, 2, 3 and 4, respectively) in serum-free medium with or without phenol red by trypan blue exclusion assay. All data shown are the mean values of three experiments ± SEM.

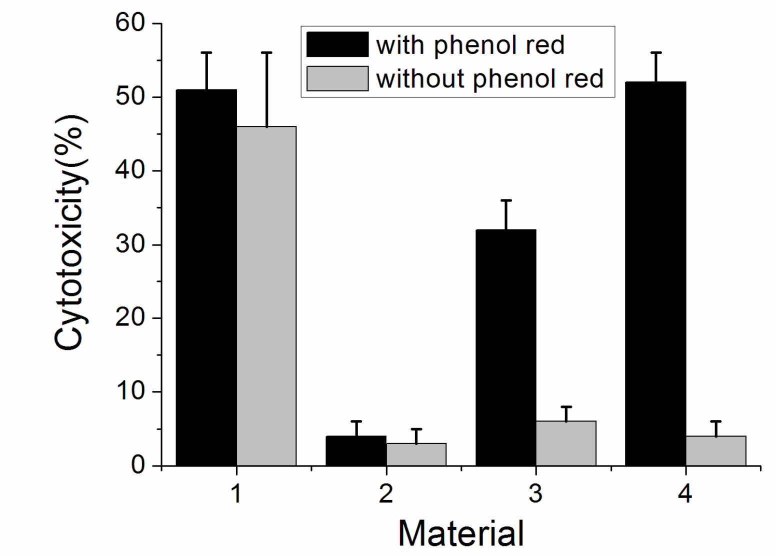

Figure S2. Cytotoxicity assessment of Hela cells exposed to MWNTs and P90 (denoted by 1 and 2, respectively) in complete cell culture medium with or without phenol red. All data shown are the mean values of three experiments ± SEM.

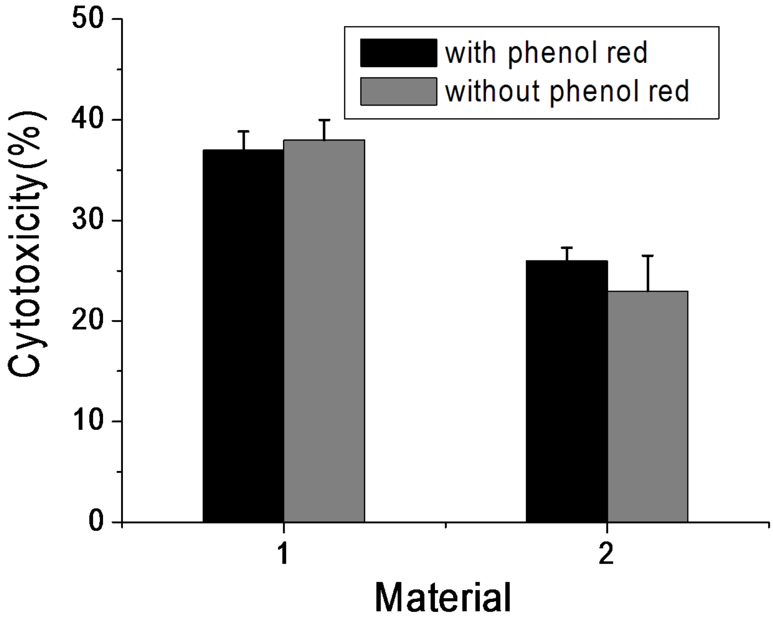

Figure S3. HPLC chromatogram of a mixture of free ^{125}I and ^{125}I-phenol red. The mobile phase was 0.1% trifluoroacetyl/acetonitrile gradient at flow rate of 1 mL/min. The UV absorption was monitored at 254 nm. Free iodine ions appeared at 2.84 minutes and ^{125}I-phenol red appeared at 13.99 minutes.

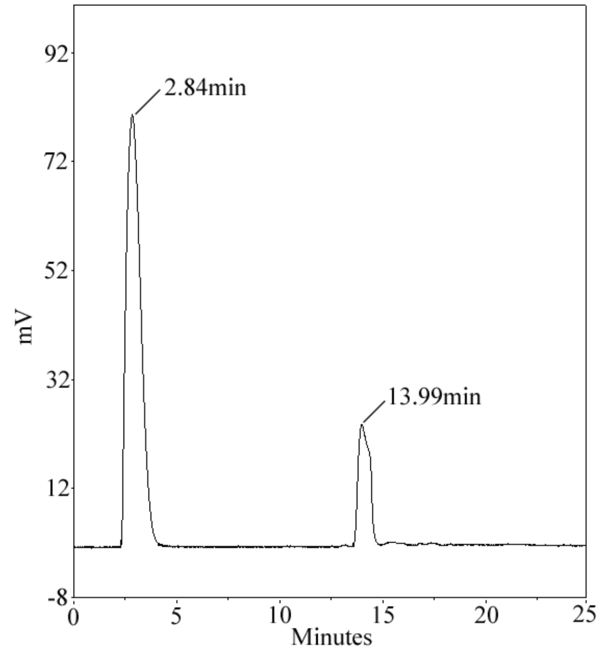

Figure S4. HPLC chromatogram of ^{125}I-phenol red. ^{125}I-phenol red appeared at 13.99 minutes with a peak area ratio of 100%.

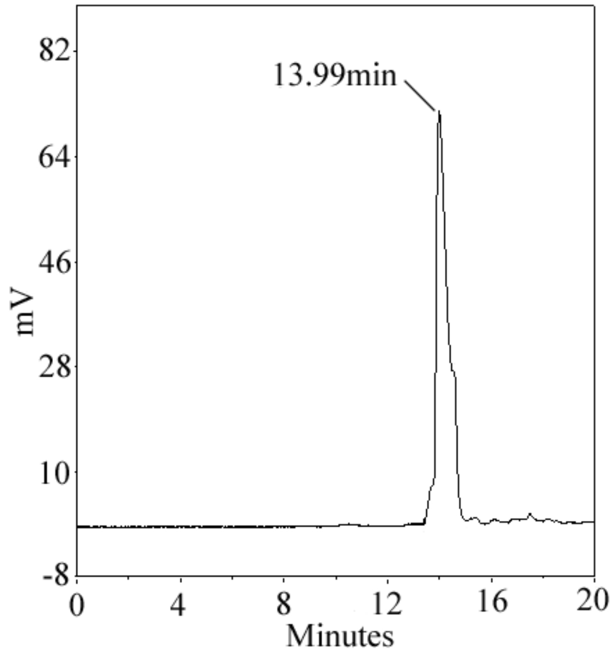

Figure S5. Standard curve of phenol red in culture medium.

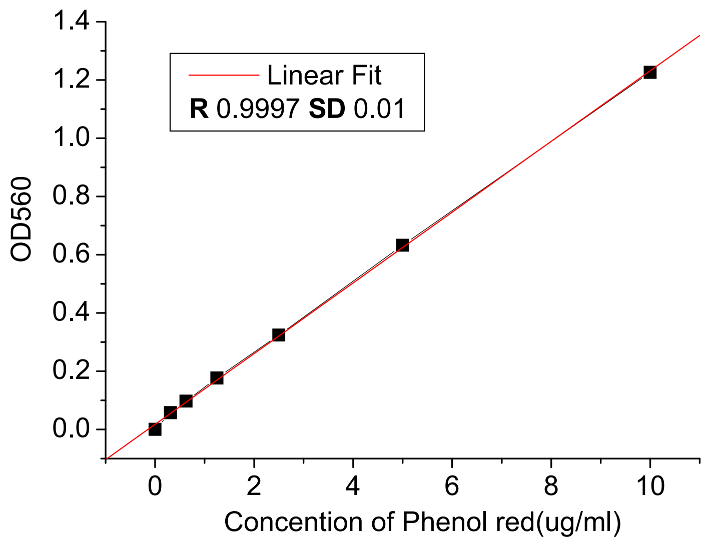

Figure S6. HPLC chromatogram of ^{125}I-phenol red after incubating in serum-free cell culture medium for 8 h. ^{125}I-phenol red appeared at 13.99 minutes with a peak area ratio of 98 % and free iodine ions appeared at 2.84 minutes with a peak area ratio of 0.5%. Im1 and Im2 are impurities.

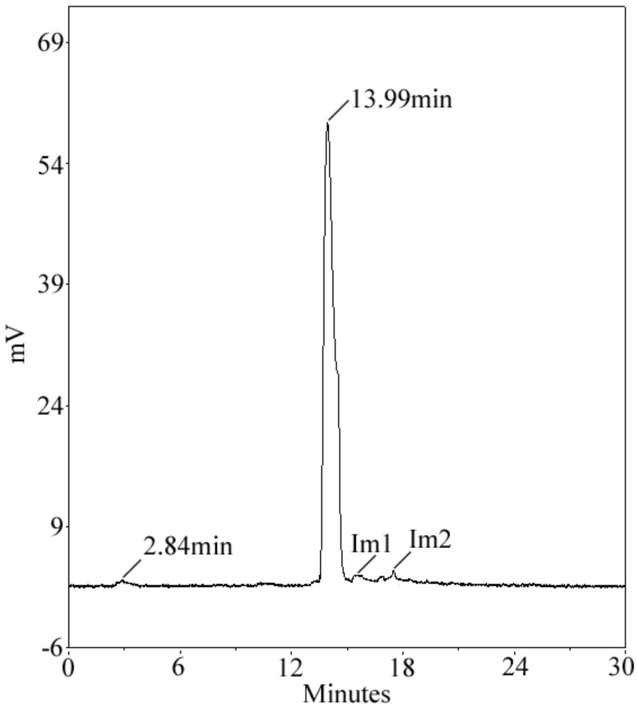

© 2012 by the authors; licensee MDPI, Basel, Switzerland. This article is an open access article distributed under the terms and conditions of the Creative Commons Attribution license (http://creativecommons.org/licenses/by/3.0/).
